# Effectiveness, efficiency, and apical extrusion of 2 rotaries and 2 reciprocating systems in removing filling material during endodontic retreatment. A systematic review

**DOI:** 10.4317/jced.59953

**Published:** 2023-03-01

**Authors:** Javier Caviedes-Bucheli, Nestor Rios-Osorio, Cristina Gutiérrez de Pineres-Milazzo, Mauricio Jiménez-Peña, Ricardo Portigliatti, Jose-Francisco Gaviño-Orduña, Marcia Antúnez-Rivero, Jose-Francisco Gomez-Sosa, Hugo-Roberto Munoz

**Affiliations:** 1 DDS, MSc, Professor and Researcher. Centro de Investigaciones Odontológicas (CIO), School of Dentistry, Pontificia Universidad Javeriana, Bogotá - Colombia; 2 DDS, MSc, Professor and Researcher. Research Department COC- CICO, Institución Universitaria Colegios de Colombia UNICOC, Bogotá, Colombia; 3 DDS. Research Department COC- CICO, Institución Universitaria Colegios de Colombia UNICOC, Bogotá, Colombia; 4 DDS, MSc, PhD, Professor and Researcher. Research Department COC- CICO, Institución Universitaria Colegios de Colombia UNICOC, Bogotá, Colombia; 5 DDS, Professor. Postgraduate Program of Endodontic Specialization Training, Buenos Aires University, Buenos Aires - Argentina; 6 DDS, PhD, Professor. School of Dentistry, University of Barcelona – Spain; 7 DDS, MSc, Professor. Department of Endodontics, Universidad Diego Portales, Santiago - Chile; 8 DDS, PhD, Professor and Researcher. Unidad de Terapia Celular, Centro de Medicina Regenerativa, Instituto Venezolano de Investigaciones Científicas, Caracas - Venezuela; 9 DDS, MSc, Professor and Researcher. Postgraduate Research Department, School of Dentistry, Universidad de San Carlos de Guatemala - Guatemala

## Abstract

**Background:**

This systematic review investigated the effectiveness, efficiency and apical extrusion of the debris of two rotary and two reciprocating single-file systems used for the removal of filling material from straight root canals.

**Material and Methods:**

A literature search was performed in the Medline, ISI Web of Science, and Scopus databases for relevant articles matching the keyword search strategy. Effectiveness was determined with studies dealing with the ability of the instruments to remove filling material from root canals. Efficiency was assessed with studies dealing with the time needed to completely remove the root canal filling, and apical extrusion was determined with studies that measured the amount of filling material extruded through the apex.

**Results:**

From the 424 articles initially found, 406 were excluded for being non-relevant or not fulfilling the selection criteria. Another 9 articles were excluded after methodology evaluation. Finally, 9 studies were included in the systematic review.

**Conclusions:**

None of the reviewed systems is effective to completely remove the filling materials from straight root canals, and all systems appear to be equally time-efficient, although this variable shows different results. In terms of apical extrusion, the analyzed reciprocating systems extrude more material toward the periapical tissues than the continuous rotation systems.

** Key words:**Systematic review, rotary files, reciprocating files, apical extrusion, endodontic retreatment.

## Introduction

Endodontic retreatment could be challenging within the endodontic practice, mainly due to difficulties for the removal of contaminated materials from the root canal system without extruding them to the periapical tissues. Therefore, several studies have been addressed to analyze the effectiveness and efficiency of endodontic instruments to achieve this purpose; as well as the periapical extrusion of debris and filling materials (1).

It is highly relevant to combine the three aforementioned variables (effectiveness, efficiency, and apical extrusion of debris) and analyze them in studies dealing with the removal of filling materials. Effectiveness is defined as the ability of an instrument to remove the filling material from the root canal system; efficiency refers to the patients’ chair-time required to perform the complete removal of the material, and apical extrusion of debris deals with the amount of material pushed by the endodontic instruments through the apical foramen into the periapical tissues during endodontic retreatment (2-4).

The main purpose of endodontic retreatment is to alleviate signs and symptoms related to infectious processes by completely removing the intra-canal filling materials from the involved root, and reduce the intra-canal bacterial load after performing an adequate protocol of cleaning and disinfection of the canal to reestablish healthy periapical tissues (5). However, it has been shown that retreatment does not guarantee total elimination of the intra-canal bacteria and that the overall reported success rate of endodontic retreatment is around 78%, in contrast to first-time conventional endodontic treatment where success range between 86% and 96% in necrotic and vital teeth, respectively (6).

To perform a more effective, efficient, and less invasive procedure in terms of apical extrusion of debris and materials, engine-driven rotatory techniques specially designed for endodontic retreatments such as Protaper Universal Retreatment system and Mtwo retreatment system have been proposed.

Recently, single-file nickel-titanium (Ni-Ti) reciprocating systems like WaveOne (Dentsply Maillefer, Ballaigues-Switzerland) and Reciproc (VDW, Munich, Germany) have been used for endodontic retreatment, even though they were not designed for such purpose. However, they have shown positive results in terms of effectiveness and efficiency during these types of procedures (7).

These system´s philosophy proposes a single-file endodontic treatment, which renders the endodontic retreatment to be time reduced. However, it has been shown that these files, are incapable of completely remove the filling material from the root canals. Moreover, it has been demonstrated that they cause greater extrusion of materials toward the periapical tissues than continuous rotation file systems (3,7).

Considering all the above, a systematic review of the literature was conducted following AMSTAR, PRISMA and Faggion guidelines (8-10), analyzing the effectiveness, efficiency and apical extrusion of two continuous rotation systems (Protaper Universal and Mtwo retreatment systems) and two single-file reciprocating systems (Wave One and Reciproc) to determine which one is the more efficient and effective and which one extrudes less amount of filling material during endodontic retreatment.

## Material and Methods

-Information sources and search strategy:

Several keywords were used for each one of the elements of interest.

1): (Retreatment OR endodontic retreatment OR endodontic failures OR endodontic retreatment success OR endodontic retreatment techniques OR filling material removal OR nonsurgical Retreatment).

2): (Rotary systems OR rotary file OR Protaper universal retreatment system OR Mtwo retreatment system);

3): (Reciprocating systems OR reciprocating instruments OR Reciproc OR WaveOne).

The electronic search of the literature has been conducted in the following databases: MEDLINE, ISI WEB OF SCIENCE and SCOPUS; using the following keyword combinations: 1 AND 2 and, 1 AND 3, and the following filters: AND humans, AND English AND 2010/01/01-2022/07/25 without filtering by journal. Table 1 shows the search strategy used for each database.

**Table 1:**
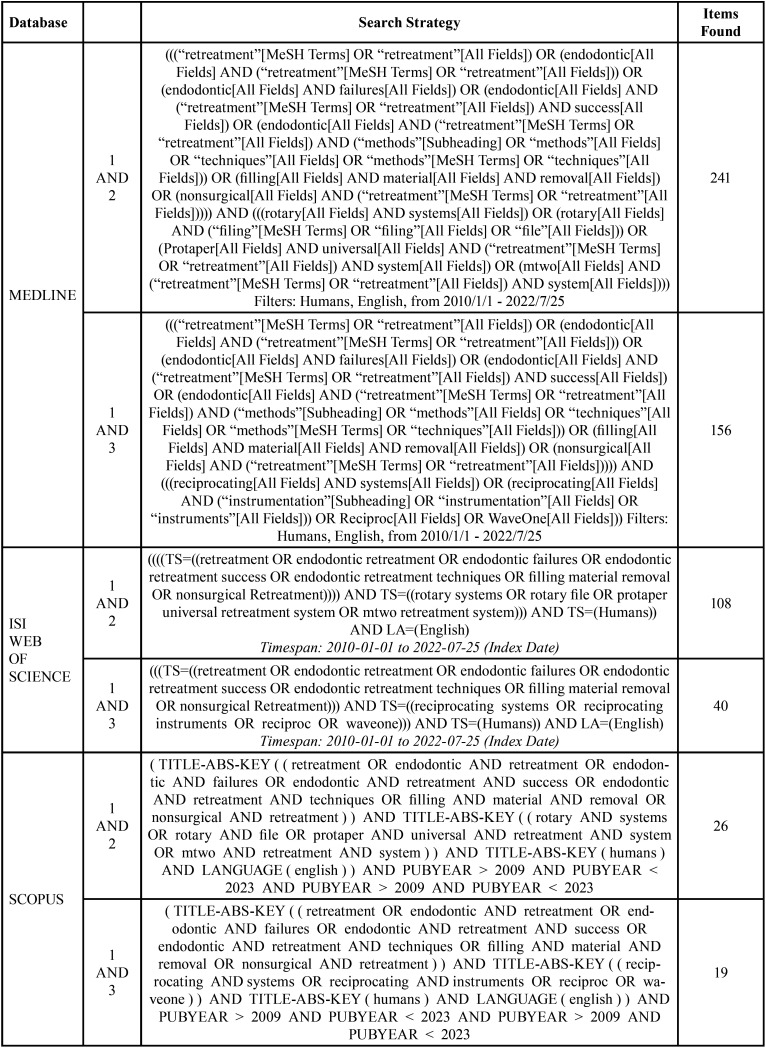
Database search strategy.

An additional manual search was also performed, searching for.

a) Titles in the bibliographic references of the selected articles, unidentified by the method described above and.

b) In-press articles in 4 high-impact Endodontic Journals (International Endodontic Journal, Journal of Endodontics, Australian Endodontic Journal and Oral Surgery Oral Medicine Oral Pathology Oral Radiology and Endodontics).

-Extraction of the information:

From the selected studies, the following criteria were extracted: Authors, name, and ranking of the journal where the study was published, title, year of publication, type of study, sample size and characteristics, randomization, biases, endodontic filling removal technique, variables, units of measurement, evaluation method, statistical analysis, level of significance and results (Table 2- Table 2 cont.-3). These data allowed submitting each study to the analysis of the methodological quality and classifying them according to the level of evidence (Table 3).

**Table 2:**
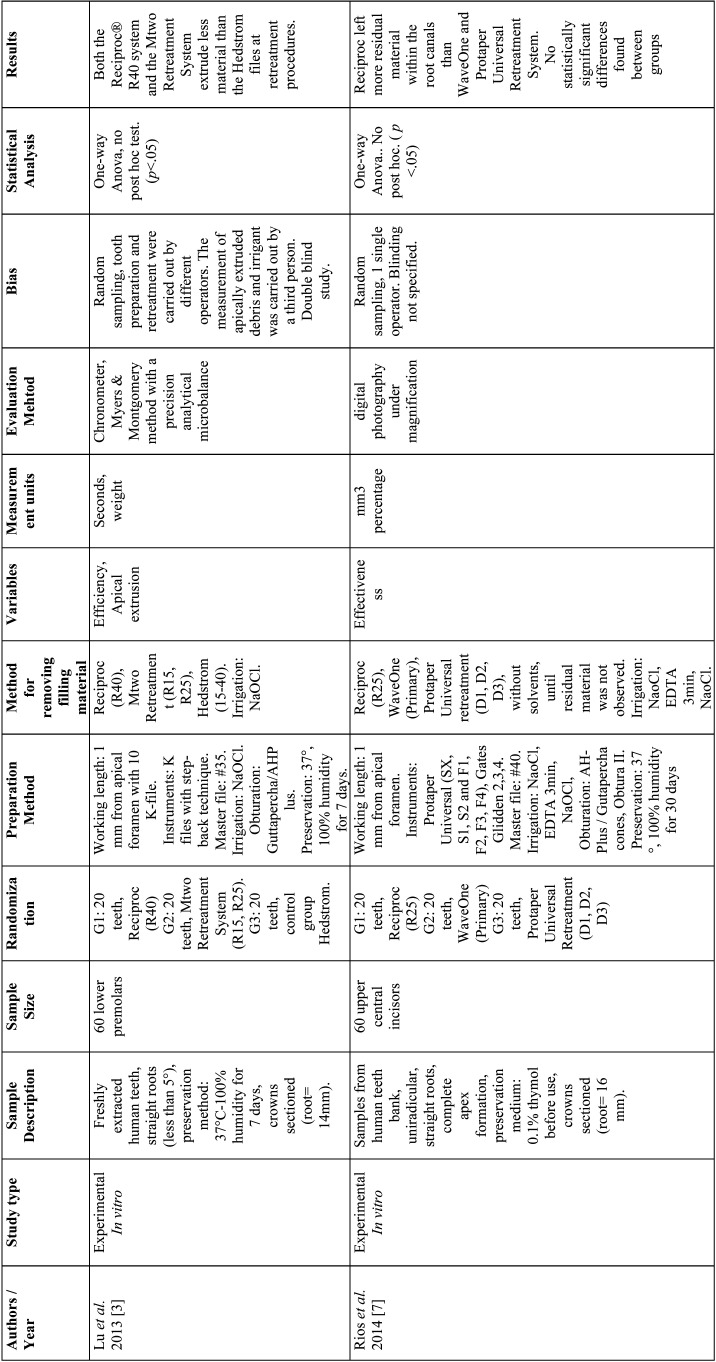
Articles included in the systematic review.

**Table 2 cont.**
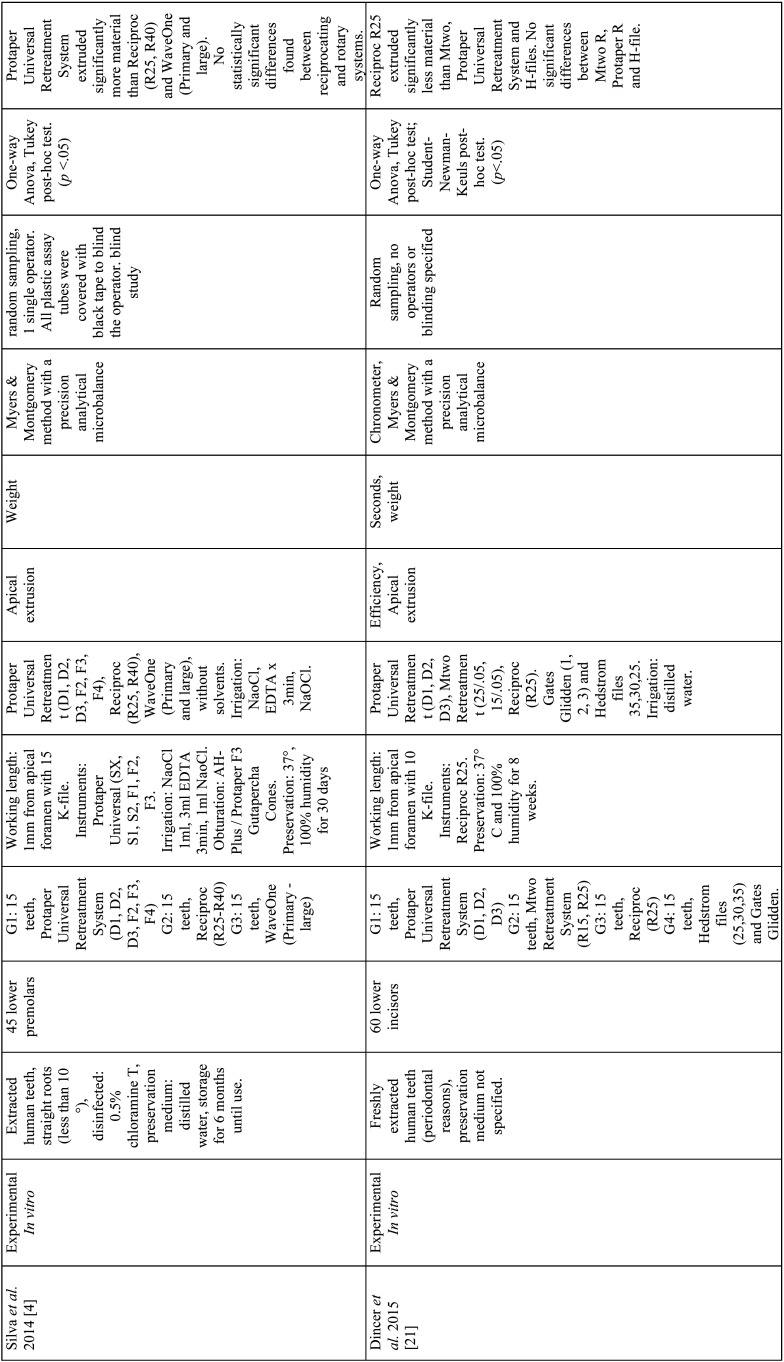
Articles included in the systematic review.

**Table 2 cont.-1:**
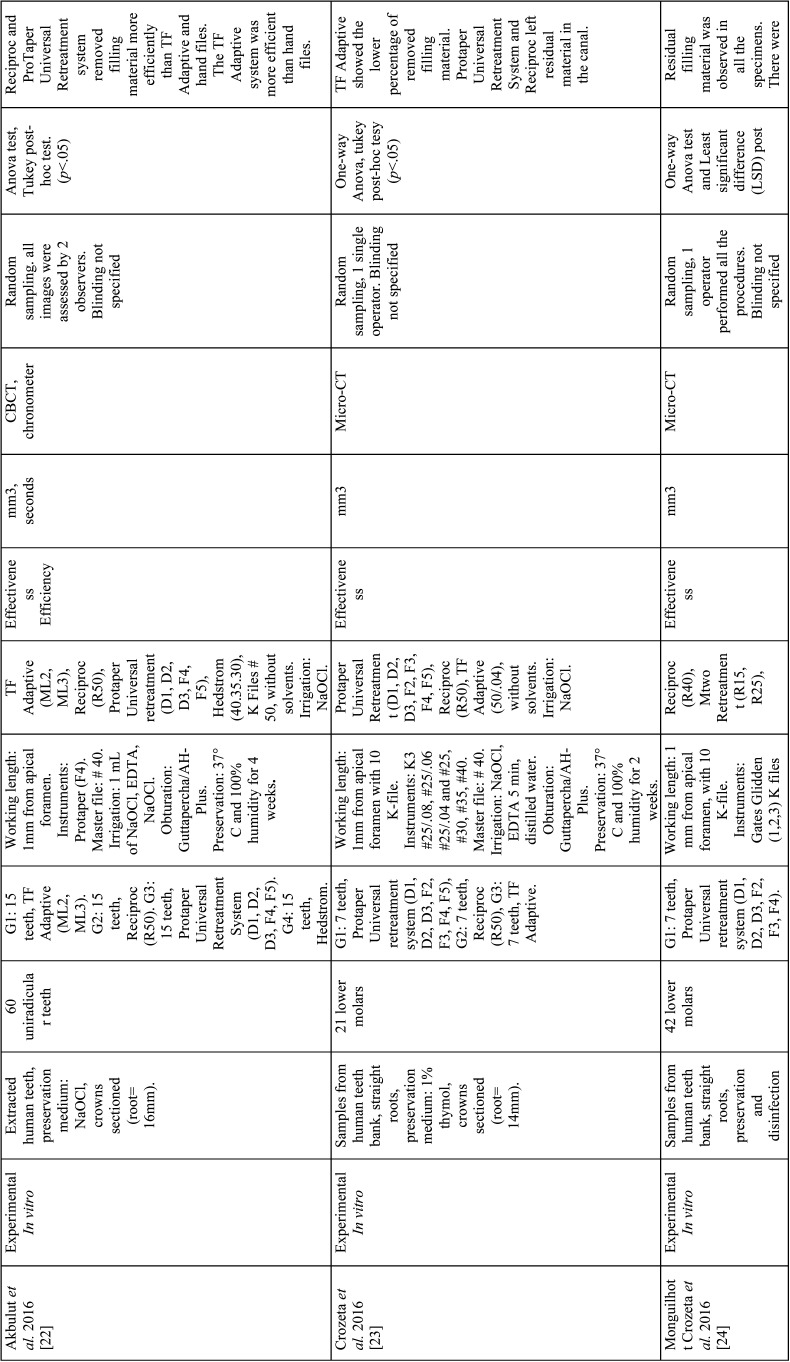
Articles included in the systematic review.

**Table 2 cont.-2:**
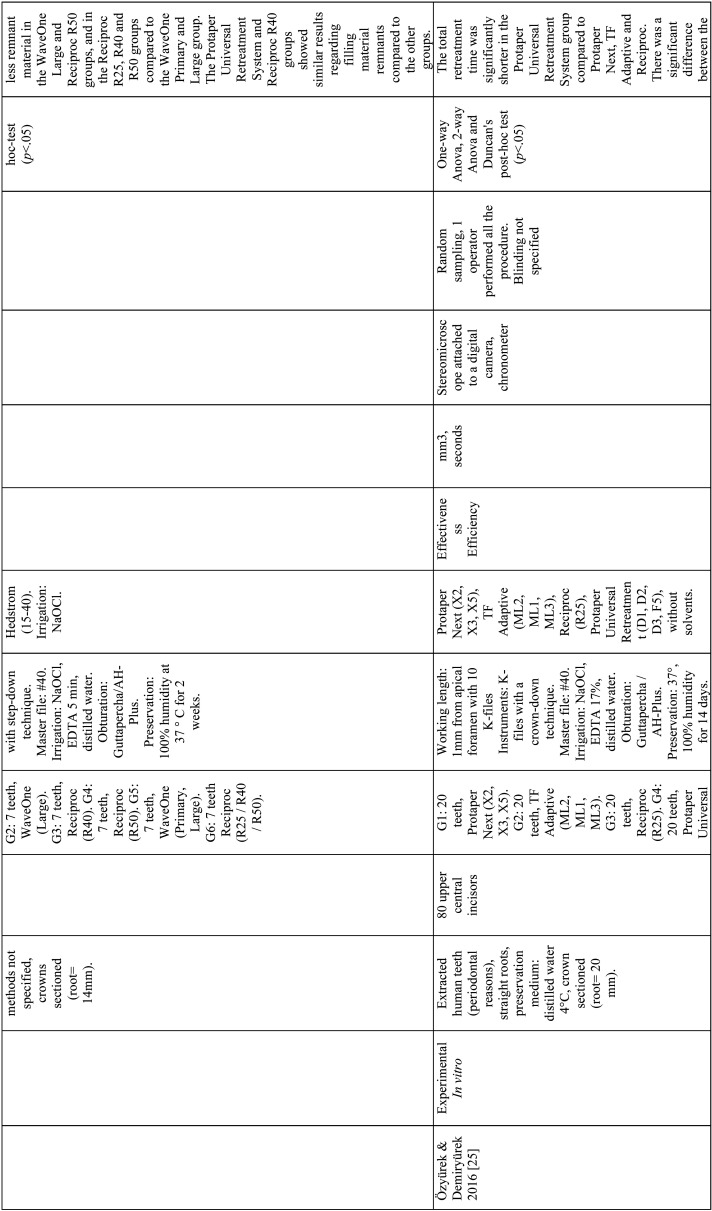
Articles included in the systematic review.

**Table 2 cont.-3:**
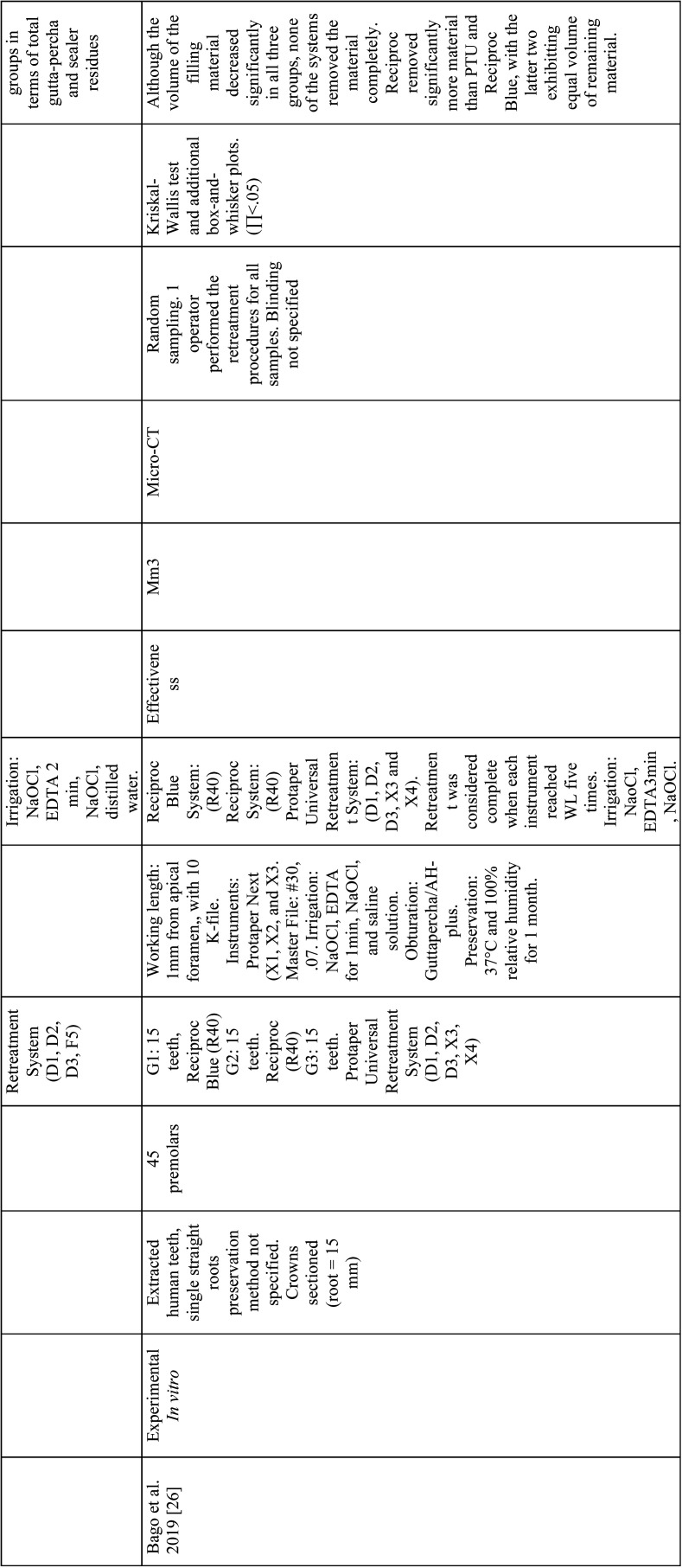
Articles included in the systematic review.

**Table 3:**
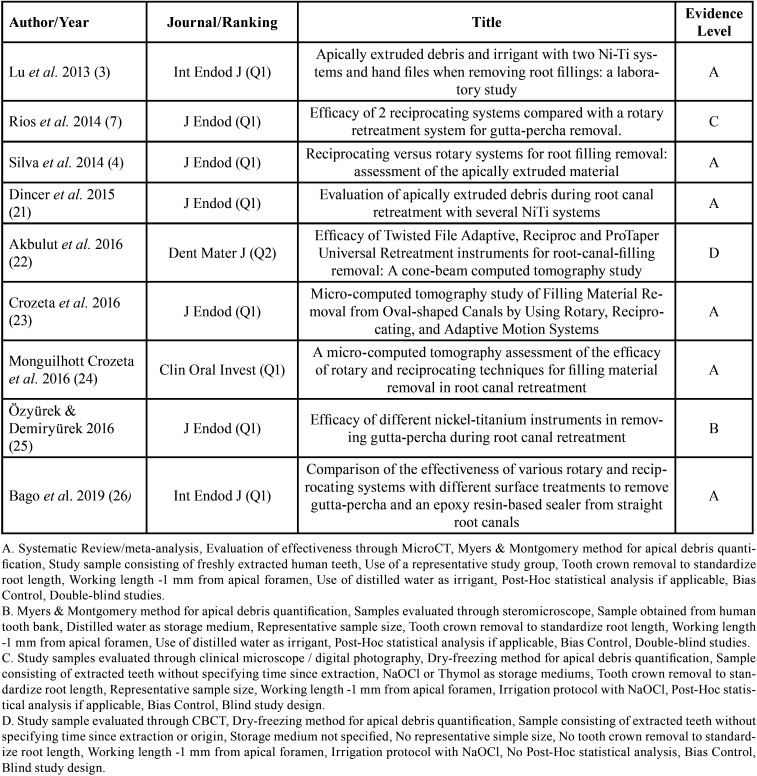
Methodological quality assessment and classification of evidence levels of the selected articles.

Results

**Figure 1:**
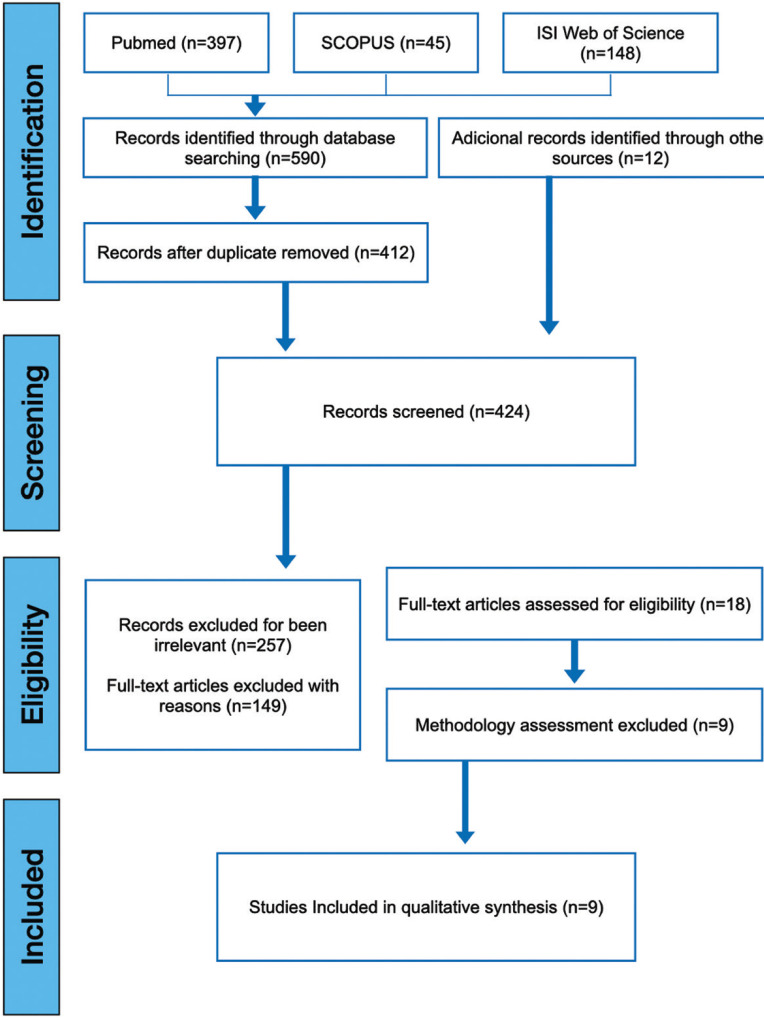
PRISMA 2020 flow diagram for new systematic reviews which included searches of databases and registers.

-Selection of the studies:

The electronic search (Fig. 1) was performed on 2022/07/25 following the flow diagram suggested by PRISMA (11). In the MEDLINE database, 241 results were obtained with the combination of criteria 1 AND 2, and 156 results with the combination of 1 AND 3. In ISI WEB OF SCIENCE, for the same combinations, the results obtained were 108 and 40 respectively. Finally, in the Scopus database, 26 and 19 results were obtained for the same combinations, for a total of 590 articles.

Removing duplicates for searches 1 AND 2 and 1 AND 3, a total of 412 articles were obtained. Additionally, 12 articles from the manual search were included for a total of 424 studies. Three reviewers (J.C., C.G., and N.R.) independently reviewed the titles, abstracts, and full-texts (in cases where there was no abstract) considering the established selection criteria (Table 4). This process allowed the exclusion of 406 articles: 257 for being irrelevant and 149 by exclusion criteria. Finally, 18 studies were chosen. In case of disagreement between the reviewers, the decisions were made by consensus. A further evaluation of the materials and methods was conducted on the selected articles, allowing the exclusion of 9 studies (12-20) due to methodological criteria (Table 5), for a final number of 9 studies selected (Fig. 1).

**Table 4:**
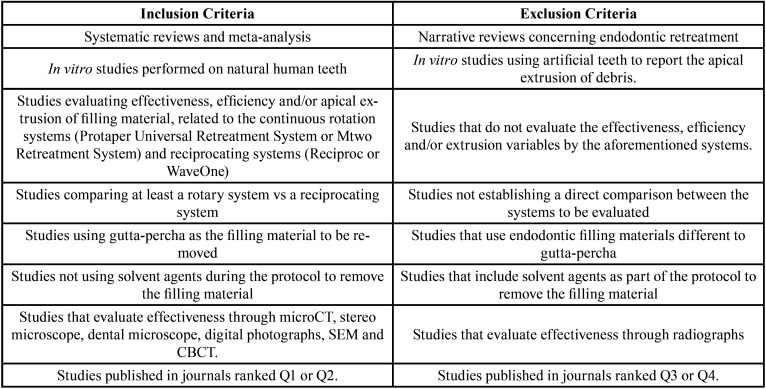
Selection Criteria.

**Table 5:**
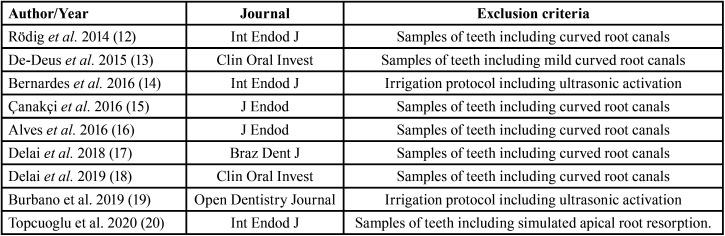
List of excluded articles after applying methodological evaluation.

A total of 9 *in vitro* articles (3,4,7,21-26) were included in the systematic review (Fig. 1). Analysis of these studies demonstrates that the evaluated systems (continuous and reciprocating) are ineffective for the complete removal of the filling material from straight root canals (23). Ríos *et al*. concluded that the WaveOne and Reciproc systems are more effective for removing the filling materials (7); while Akbulut *et al*. concluded that there is no statistically significant difference between Protaper Universal Retreatment system and Reciproc (22). Moreover, Monguilhott Crozeta *et al*. reported that the Protaper Universal Retreatment system and Reciproc displayed similar volumes of residual material within the analyzed root canals (24).

Regarding the efficiency of the systems to remove the filling material from straight root canals, Özyürek & Demiryürek found that the Protaper Universal Retreatment system is significantly faster and even more effective than the Reciproc system (25). However, Dincer *et al*. concluded that the Protaper Universal Retreatment system and Reciproc are equally efficient and both require less time to remove gutta-percha than the Mtwo retreatment system (21).

Finally, regarding apical extrusion of filling material, Lu *et al*. concluded that Reciproc extrudes more material than the Mtwo Retreatment system, since continuous rotation promotes the transport of the endodontic materials toward the coronal tooth portion, while the reciprocating movement favors the instrument to push the filling material residues toward the apex (3). Likewise, the study by Silva *et al*. reported that there are no differences between Reciproc and WaveOne in terms of apical extrusion of filling materials and debris and that the apical extrusion of material occurs independently of the retreatment system used, as all of them produce at least a minimum of material and debris extrusion toward the apex (4).

## Discussion

This systematic review evaluates the effectiveness, efficiency and the apical extrusion of filling material of two rotary retreatment systems (Protaper Universal Retreatment and Mtwo retreatment systems) compared to two reciprocating systems (Reciproc and WaveOne), for removing gutta-percha from straight root canals. These systems were selected because there are not enough studies published from other retreatment systems to analyze the data under similar conditions.

In vitro research on natural teeth evaluating new materials and techniques for subsequent *In vivo* human use is an important issue of modern dentistry since this type of studies can be conducted under controlled conditions; therefore, In vitro studies conducted on natural human teeth were established as essential inclusion criteria, although *in vitro* studies cannot reproduce a dynamic clinical setting (10). The use of freshly extracted human teeth allows operators to simulate clinical endodontic procedures as close as possible to reality (7), and considering several aspects such as similarities regarding clinical environment conditions, hardness, elasticity, humidity, and natural consistency of the dentin, allow achieving a well-structured analogy of the management of human root canals (27).

Storage and preservation solutions of tooth samples is a transcendental variable to be taken into account at *in vitro* research, as these solutions are needed to preserve and retain the physical, chemical, and mechanical properties of the stored teeth, and not to alter and/or influence the results of the studies. Among the most used and effective storage solutions are distilled water and chloramine T, since they do not generate changes on enamel or the dentin structure (28).

The studies included in the systemic review have similar purposes and methodologies, evidencing the existence of comparable study groups and similar tooth selection parameters for evaluating the effectiveness, efficiency, and apical extrusion variables. Only studies with straight roots samples were taken into account to increase the probability that such variables (effectiveness, efficiency, and apical extrusion) were analyzed only under the instruments used criteria and were not influenced by root morphology. Inclusion of studies with curved canals would difficult to reach valid conclusions, as they were not performed in similar conditions, following the quality standards guidelines from Faggion, PRISMA, and AMSTAR for selecting studies to be included in a systematic review (8-10).

The working length has also been taken into consideration as it may influence the amount of apically extruded debris. It has been previously shown that a working length of −1 mm from the apical foramen significantly reduces debris extrusion (29), and that, when working on extracted teeth, there is a lack of apical resistance which is naturally provided by the periapical tissues, and therefore, a working length shorter than 1 mm from apical foramen could favor apical debris extrusion (30,31).

Another criterion to consider is the tooth crown sectioning in the selected studies, to standardize the working length and the approximate amount of filling material within the samples, and to rule out the influence of variables such as the crown anatomy and the access to the root canal, thus resulting in more reliable studies (32-34).

The irrigant solutions used during the removing filling material procedure in the selected studies were distilled water (21) and sodium hypochlorite (3,4,7,22-25). The use of distilled water as an irrigant avoids any increase in the weight of the samples, as the formation of sodium hypochlorite crystals has been reported after the evaporation of the extruded liquid (29,35). However, other authors used sodium hypochlorite to make the study similar to clinical conditions (36). In terms of effectiveness and efficiency, the irrigant solutions did not influence variable measurement.

Regarding the inclusion criteria, only studies using gutta-percha as filling material have been selected, as it has been widely demonstrated that besides being the most used root canal filling material, it is ideal to accomplish a three-dimensional seal of the root canal space, preventing bacterial re-infection by blocking the passage of microorganisms and toxins to the periapical tissues. Gutta-percha has been proven to be a highly biocompatible material, with desirable physical and mechanical properties such as dimensional stability and easy insertion and removal from the root canal system (37).

Exclusion criteria were applied to studies using solvent agents during the process of the filling material removal. Although solvent-softened gutta-percha greatly simplifies the removal of filling materials, it may also produce a residual film of softened material along the dentinal walls of the canal, which could affect the procedure´s efficacy (2,32,38). Alternatively, chloroform-based solvents are highly cytotoxic when reaching periapical tissues, therefore its use at the root apical third is not recommended (7).

One of the techniques with the highest levels of scientific evidence to quantify the residual filling material is the micro-computed tomography (micro-CT). The micro-CT imaging offers a noninvasive and reproducible high-resolution technique for a 3-dimensional (3D) quantitative evaluation of filling materials (in mm3) before and after instrumentation, allowing a highly accurate calculation of the percentage of residual filling material left inside the root canals after retreatment (12).

Longitudinal sectioning of samples before stereomicroscope analysis to evaluate the presence of root canals filling material remnants has also been proposed (39,40). This methodology is effective in measuring remaining filling material when combined using the dental microscope and photographic analysis to obtain clinical images. Nevertheless, the method of sectioning the tooth with stainless steel disks must be performed with high precision to avoid removing the gutta-percha remains, thereby modifying and altering the study samples (41).

Cone-Beam computed tomography (CBCT) is an easily applicable noninvasive clinical tool that provides 3D imaging and quantitative evaluation that could be another eligible method for retreatment evaluation. However, root filling materials are usually radiopaque and may cause artifacts on the CBCT images, although these artifacts can be reduced with proper machine settings and parameters. Smaller voxel sizes and small FOV scans are preferable to minimize the presence of artifacts (22). These artifacts yield discrepancies in the reconstructed images and may lead to misinterpretations affecting the veracity of the study (42). Therefore, CBCT studies were qualified with a lower evidence level method since results are not completely reliable.

This systematic review did not consider studies that evaluate effectiveness through radiographic images, since radiographic images provide only two-dimensional information of a three-dimensional structure, and may be subjected to distortions, which could affect the veracity of the studies (43).

Regarding apical extrusion, the rotary systems still extrude a quantity of material and debris toward the periapical tissues, even though it has been proposed that continuous rotation movement allows the filling material to be dragged coronally (3). Although single-file reciprocating systems are time-efficient at the removal of gutta-percha during endodontic retreatment, it has been shown that they may extrude a greater amount of filling material and debris toward the apex, due to the dynamic of the alternating movement (reciprocating movement), which is a very aggressive movement that removes a large amount of material in a short time, pushing endodontic material and debris toward the apex (41).

Another important inclusion criteria for study selection was the use of the Myers & Montgomery technique or the dry-freezing technique for measuring the amount of extruded filling material. However, the Myers & Montgomery technique has more advantages than the second one as it allows a separate quantification of the amount of extruded material and the quantity of irrigant (44).

Considering the results of the included studies, it is concluded that none of the systems is capable of completely removing the filling material from root canals. This finding is reported in the literature as the impossibility of removing 100% of the total filling material regardless of the applied technique (2,45). However, although the Reciproc and WaveOne systems were not designed specifically for endodontic retreatment, it may be inferred that their special design in conjunction with the reciprocating movement could benefit the material removal, not presenting significant differences regarding effectiveness when compared to Protaper Universal retreatment system (7). In terms of efficiency, there are some different results among the scientific literature that may be related to several variables such as variables inherent to the operator and methods used to calculate the total time required for gutta-percha removal (40).

## Conclusions

None of the reviewed systems is effective to completely remove the filling materials from straight root canals, and all systems appear to be equally time-efficient, although this variable shows different results. In terms of apical extrusion, the analyzed reciprocating systems extrude more material toward the periapical tissues than the continuous rotation systems.
